# Efficacy of early intervention on the bowel damage and intestinal surgery of Crohn’s disease, based on the Lémann index

**DOI:** 10.1186/s12876-020-01575-7

**Published:** 2020-12-11

**Authors:** Mingming Zhu, Qi Feng, Xitao Xu, Yuqi Qiao, Zhe Cui, Yunqi Yan, Zhihua Ran

**Affiliations:** 1 Division of Gastroenterology and Hepatology, Key Laboratory of Gastroenterology and Hepatology, Ministry of Health, Shanghai Inflammatory Bowel Disease Research Center, Renji Hospital, School of Medicine, Shanghai Jiao Tong University, Shanghai Institute of Digestive Disease, Shanghai, China; 2 Department of Radiology, Renji Hospital, School of Medicine, Shanghai Jiao Tong University, Shanghai, China; 3 Department of Gastrointestinal Surgery, Renji Hospital, School of Medicine, Shanghai Jiao Tong University, Shanghai, China

**Keywords:** Early crohn’s disease, Infliximab, Lémann index, Bowel damage, Intestinal surgery

## Abstract

**Background:**

Clinicians aim to prevent progression of Crohn’s disease (CD); however, many patients require surgical resection because of cumulative bowel damage. The aim of this study was to evaluate the impact of early intervention on bowel damage in patients with CD using the Lémann Index and to identify bowel resection predictors.

**Methods:**

We analyzed consecutive patients with CD retrospectively. The Lémann Index was determined at the point of inclusion and at follow-up termination. The Paris definition was used to subdivide patients into early and late CD groups.

**Results:**

We included 154 patients, comprising 70 with early CD and 84 with late CD. After follow-up for 17.0 months, more patients experienced a decrease in the Lémann Index (61.4% vs. 42.9%), and fewer patients showed an increase in the Lémann Index (20% vs. 35.7%) in the early compared with the late CD group. Infliximab and other therapies reversed bowel damage to a greater extent in early CD patients than in late CD patients. Twenty-two patients underwent intestinal surgery, involving 5 patients in the early CD group and 17 patients in the late CD group. Three independent predictors of bowel resection were identified: baseline Lémann index ≥ 8.99, disease behavior B1, and history of intestinal surgery.

**Conclusions:**

Early intervention within 18 months after CD diagnosis could reverse bowel damage and decrease short-term intestinal resection. Patients with CD with a history of intestinal surgery, and/or a Lémann index > 8.99 should be treated aggressively and monitored carefully to prevent progressive bowel damage.

## Background

Crohn’s disease (CD) is a chronic, destructive, and progressive disease of the gastrointestinal tract. In recent years, the prevalence of CD has increased in both Asian and Western countries [1-3]. Current data shows that in China, the estimated incidence of CD is 0.51–1.09 cases per 100,000 persons [4, 5].

At diagnosis, most patients with CD show chronic inflammatory behavior [6]. However, during the course of the disease, CD can cause structural bowel damage (BD) over time, such as fibrostenotic or penetrating complications [7, 8]. Preventing the progression of BD has become a key goal to improve the long-term prognosis of patients with CD [9].

Recently, to quantify and measure cumulative BD in patients with CD, the Lémann index (LI), which is based on small bowel imaging, endoscopy, previous surgery and perianal assessment, was developed [10]. The LI has been used to evaluate the progression of CD and the efficacy of treatment [11, 12].

Earlier use of biological agents tended to slow down the progression of BD [13]. Targeting early CD might be the best way to change the disease course and maximize patient benefit. Unfortunately, patients with CD often experience a delay in diagnosis, which is associated with an increased risk of BD over time [7, 8]. The Paris definition describes early CD as having a disease duration less than or equal to 18 months, without a history of previous treatment using disease-modifying agents (e.g., biologics, immunomodulators) [14]. Among the anti-tumor necrosis factor (TNF) agents currently available to treat CD, only infliximab (IFX) is approved in China. However, data supporting the effectiveness of IFX on BD in patients with CD are limited in China, because of the lower infusion rate of IFX and lower incidence of CD. Therefore, the main objective of this study was to determine the effect of early intervention on short-term outcomes of BD using LI, based on magnetic resonance enterography (MRE), and to identify predictors of short-term bowel resection in a series of patients with CD.

## Methods

### Participants and protocols

In our center, we conducted a retrospective, single-center study in patients with CD between July 2013 and October 2018. Criteria for inclusion in this study were patients older than 16 years and were diagnosed with CD according to histological, endoscopic, clinical, and pathological examinations. Patients involved in the study underwent MRE and endoscopy and/or pelvic magnetic resonance imaging (MRI) after surgical drainage if needed, within 4 weeks at baseline and at the end of follow-up. Different treatments were selected by the physician according to the patients’ condition, including infliximab (5 mg/kg at weeks 0, 2, and 6, followed 5 mg/kg every 8 weeks), azathioprine (1.5 or 2.0 mg/kg/d) combined with steroids, [15] other immunomodulators (tacrolimus or methotrexate), enteral nutrition, or mesalazine (3.0–4.0 mg/day) therapy.

The exclusion criteria included aged < 16 years, absence of two serial MRE data, incomplete follow-up data, history of treatment with biological agents within 12 months, history of intestinal surgery within 12 months, and less than 12 months’ of follow‐up.

The patients were subdivided into an early CD group and a late CD group according to the Paris definition. The early CD group comprised patients whose disease duration was less than or equal to 18 months and who had no previous history of the use of disease-modifying agents, which is independent of BD [14]. The remaining patients whose disease duration was longer than 18 months and/or had started treatment with disease-modifying agents were defined as the late CD group.

From the patients’ medical records, the following data were extracted: Age; sex; smoking history; disease duration; age at CD diagnosis; age at enrollment; basic laboratory tests; clinical disease activity scores (CDAI); previous medical and surgical interventions; and time between initial and follow-up MRE (months).

### Calculation of Lémann index

For each patient, MRE-based LIs were calculated by scoring the following factors: endoscopy data, previous surgery, extension, location, and intestinal complications, according to Pariente et al. [10] Endoscopic data, MRE of the small bowel, and pelvic MRI were reviewed by senior radiologists and gastroenterologists who were blinded to the other procedures of this study and had more than 10 years of experience.

The LI score was calculated by conceptually dividing the gastrointestinal tract into four segments. 1) The upper digestive tract, comprising the duodenum, stomach, and esophagus; 2) the small bowel tract, which was further subdivided into 20 segments each of 20-cm in length; 3) the colon and rectum, including sigmoid colon, descending colon, transverse colon, ascending colon, cecum, and rectum; 4) the anus. Investigators scored the information on previous operations, stricturing and/or penetrating lesions of maximal severity for each segment (grades 1–3). A known coefficient for each segment and the overall level of organ damage were calculated [10].

Patients whose LI was unchanged were defined as “stabilized”, those with a decreased LI as “Improved”, and those with an increased LI as “Deteriorated”.

### Statistical analysis

To summarize continuous variables, medians with the interquartile range (IQR) were used. For discrete data, percentages were computed. A chi-squared test or Fisher's exact test were used to compare categorical variables, and the Mann–Whitney U-test was used to compare the differences between independent groups. To determine the ideal cut-off value of LI to predict the risk factors of bowel surgery, analysis using receiver operating characteristic (ROC) curves analysis was used. We also calculated the area under the curve (AUC) with 95% confidence intervals (CIs), sensitivity, and specificity. Univariate logistic regression was performed to identify significant predictors of increase in short-term intestinal resection. Multivariate analysis was then performed on the variables with a *P* value < 0.10 from the univariate analysis. IBM SPSS Statistics 21.0 software (IBM Corp., Armonk, NY, USA) was used to perform all the statistical analyses. Statistical significance was accepted at a two-sided *P *value of < 0.05.

## Results

### Baseline characteristics of the patients

A total of 209 patients with data from at least two serial MREs and endoscopic data were potentially eligible. Finally, 154 patients with at least two serial MRE examinations met the inclusion criteria and were included, whereas 55 patients were excluded (aged < 16 years, n = 8, MRE performed less than 12 months apart, n = 21, incomplete follow-up data, n = 15, history of intestinal surgery within 12 months, n = 11).

The baseline characteristics of the included patients are summarized in Table 1. Approximately two-thirds of the population was male. Seventy (45.5%) patients received an early CD diagnosis. Sixty-nine (44.8%) patients had perianal involvement in our IBD center, stabilized.

**Table 1:** Demographic and clinical characteristics-grouped by disease duration

Variable	Total patients (n = 154)	Early CD(n = 70)	Late CD(n = 84)	*P* value
Gender(male: female)	103: 51	44: 26	58: 26	0.42
Median age on set of CD, years	24.5 [17.0–29.0]	25.0 [17.0–28.0]	24.0 [15.5–31.0]	0.56
Median age at enrollment, years	29.0 [23.0–36.0]	26.0 [19.8–30.5]	33.0 [27.00–39.8]	< 0.001
Median disease duration, years	4.5 [1.0–7.0]	1.0 [0.5–2.0]	7.0 [4.0–9.0]	< 0.001
Montreal (Age)				
A1 (16 years or younger)	16 (10.4%)	8 (11.4%)	8 (10.7%)	0.43
A2 (17‐40 years)	114 (74.0%)	53 (75.7%)	61 (72.6%)	0.42
A3 (more than 40 years)	24 (15.6%)	9 (12.9%)	15 (17.9%)	0.36
Montreal L (location)				
L1 (ileal)	72 (47.1%)	38 (54.3%)	32 (38.1%)	0.43
L2 (colonic)	9 (5.8%)	3 (4.3%)	6 (7.1%)	0.19
L3 (ileocolonic)	71 (46.1%)	26 (37.1%)	45 (53.6%)	0.16
L4 (upper GI)	31 (20.1%)	13 (18.6%)	19 (22.6%)	0.60
Montreal B (Behavior)				
B1	94 (61.0%)	46 (65.7%)	48 (57.1%)	0.26
B2	40 (26.0%)	17 (24.3%)	23 (27.4%)	0.51
B3	26 (16.9%)	9 (12.9%)	17 (20.2%)	0.53
Perianal disease	69 (44.8%)	37 (52.9%)	32 (38.1%)	0.07
Previous surgical resection	27 (17.5%)	7 (10.0%)	20 (23.8%)	0.02
Previous medical treatments				
Steroids	24 (15.6%)	9 (12.9%)	15 (17.9%)	0.08
Immunomodulators	43 (27.9%)	0 (0%)	43 (51.2%)	< 0.001
5-ASA	41 (26.7%)	18 (25.7%)	23 (27.4%)	0.62
Smoking habit	12 (7.8%)	4 (5.7%)	8 (9.5%)	0.23
Baseline serological markers				
CRP, mg/L	4.3 [0.5–15.3]	6.16 [0.4–24.9]	5.2 [0.5–13.1]	0.12
ESR, mm/h	22.0 [12.0–45.0]	19.0 [12.0–42.0]	24.0 [13.0–49.0]	0.10
ALB, g/L	38.6 [31.5–43.6]	38.7 [32.1–42.6]	38.4 [30.6–44.7]	0.70
HB, g/L	125.0 [101.0–141.3]	126.5 [105.0–140.3]	124.0 [95.25–142.0]	0.24
PLT, × 10^9/L	240.0 [196.0–314.3]	247.0 [197.0–342.0]	229.0 [193.8–297.3]	0.12
Current serological markers				
CRP, mg/L	0.6 [0.2–3.0]	0.55 [0.2–2.2]	0.57 [01.7–3.4]	0.61
ESR, mm/h	14.0 [3.0–22.0]	12.0 [2.0–20.0]	15.0 [3.0–24.0]	0.16
ALB, g/L	42.0 [32.7–46.0]	42.6 [33.4–46.3]	41.05 [30.28–45.6]	0.27
HB, g/L	132.0 [113.0–147.0]	136.0 [122.0–149.3]	126.5 [111.0–146.8]	0.27
PLT, × 10^9/L	225.5 [199.0–286.0]	225.0 [198.0–283.0]	228.0 [199.0–300.0]	0.73
Inclusion LI	6.2 [2.4–10.7]	6.3 [1.7–10.5]	5.9 [3.0–11.0]	0.67
Follow up LI	4.5 [1.7–9.4]	3.4 [1.6–7.5]	5.2 [2.0–10.5]	0.02
Follow up time (months)	17.0 [13.2–23.3]	15.0 [13.0–25.0]	18.5 [13.5–24.0]	0.18
CD-related bowel surgery	22 (14.3%)	5 (7.1%)	17 (20.2%)	0.001
Stabilized	31 (20.1%)	13 (18.6%)	18 (21.4%)	0.29
Improved	79 (51.3%)	43 (61.4%)	36 (42.9%)	< 0.001
Deteriorated	44 (28.6%)	14 (20%)	30 (35.7%)	< 0.001

Data are expressed as number (%) or median[IQR]

B1, nonstricturing nonpenetrating; B2, stricturing; B3, penetrating; 5-ASA, 5-aminosalicylic; CRP, C-reactive protein; ESR, erythrocyte sedimentation; ALB, Albumin; HB, Hemoglobin; PLT, Platelet; LI, Lémann index. Patients whose LI was unchanged were defined as “stabilized”, those with a decreased LI as “Improved”, and those with an increased LI as “Deteriorated”

On the basis of the Paris definition, the study population was subdivided the early CD group (n = 70, 45.5%), and the late CD group (n = 84, 54.5%). In terms of median follow-up time, there was no statistical difference between two groups (*P* = 0.18). The median disease duration in the two groups were 1.0 year [0.5–2.0] and 7.0 years [4.0–9.0], respectively, *P* < 0.001. The two groups showed a significant difference in terms of median age at enrollment (26.0 years [19.8–30.5] vs. 33.0 years [27.0–39.8], *P* < 0.001), whereas between the two groups, there was no statistically significant difference for median age on set of CD, disease location, perianal involvement, and behavior. (Table 1).

### Comparison of the Lémann Index in patients with different disease durations

At the time of inclusion, the median LI was calculated as 6.2 [2.4–10.7]. There was no significant difference in the LI score between the early CD group (6.3 [1.7–10.5]) and the late CD group (5.9 [3.1–11.0], *P* = 0.67).

**Fig. 1:**
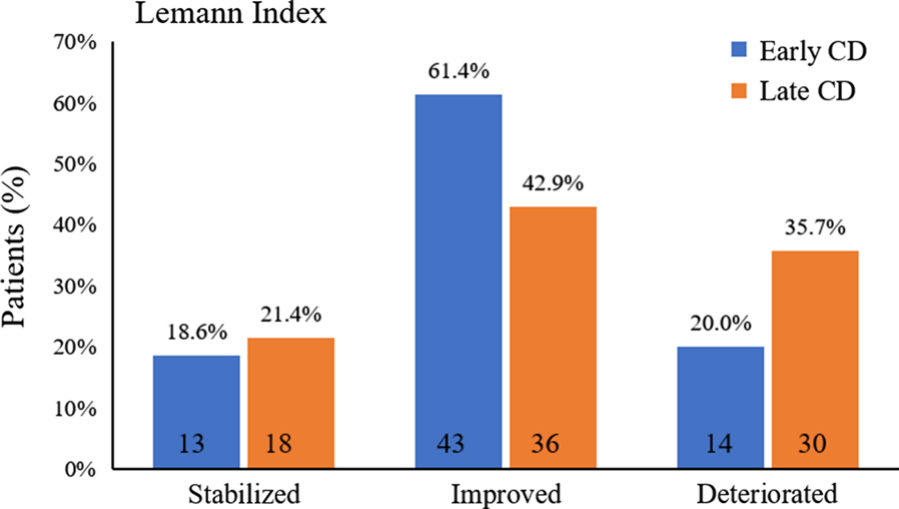
The changes of Lémann index (LI) at follow-up termination in the early Crohn’s disease (CD) and late CD groups. Patients whose LI was unchanged were defined as “stabilized”, those with a decreased LI as “Improved”, and those with an increased LI as “Deteriorated”

Among the 154 patients, at the end of follow-up, the LI decreased to a median of 4.5 [1.7–9.4], *P* = 0.001. The LI score decreased in 79 (51.3%) patients, remained unchanged in 31 (20.1%) patients, and increased in 44 (28.6%) patients. When subgroup analysis was performed, we found that the median LI score decreased significantly from 6.3 [1.7–10.5] to 3.4 [0.6–7.5] in the early CD group (*P* = 0.001), whereas it remained unchanged in the late CD group (*P* = 0.34). At follow up termination, 61.4% patients and 42.9% patients experienced a decrease in LI in the early and late CD group, respectively, *P* < 0.001. 20.0% patients and 35.7% patients showed an increased LI score, respectively, *P* < 0.001, whereas there was no significant difference in proportion of patients with a stable LI score between the two groups (18.6% vs. 21.4%), *P* = 0.29. (Fig. 1).

### Comparison of the effect of different therapies on the Lémann index

**Fig. 2:**
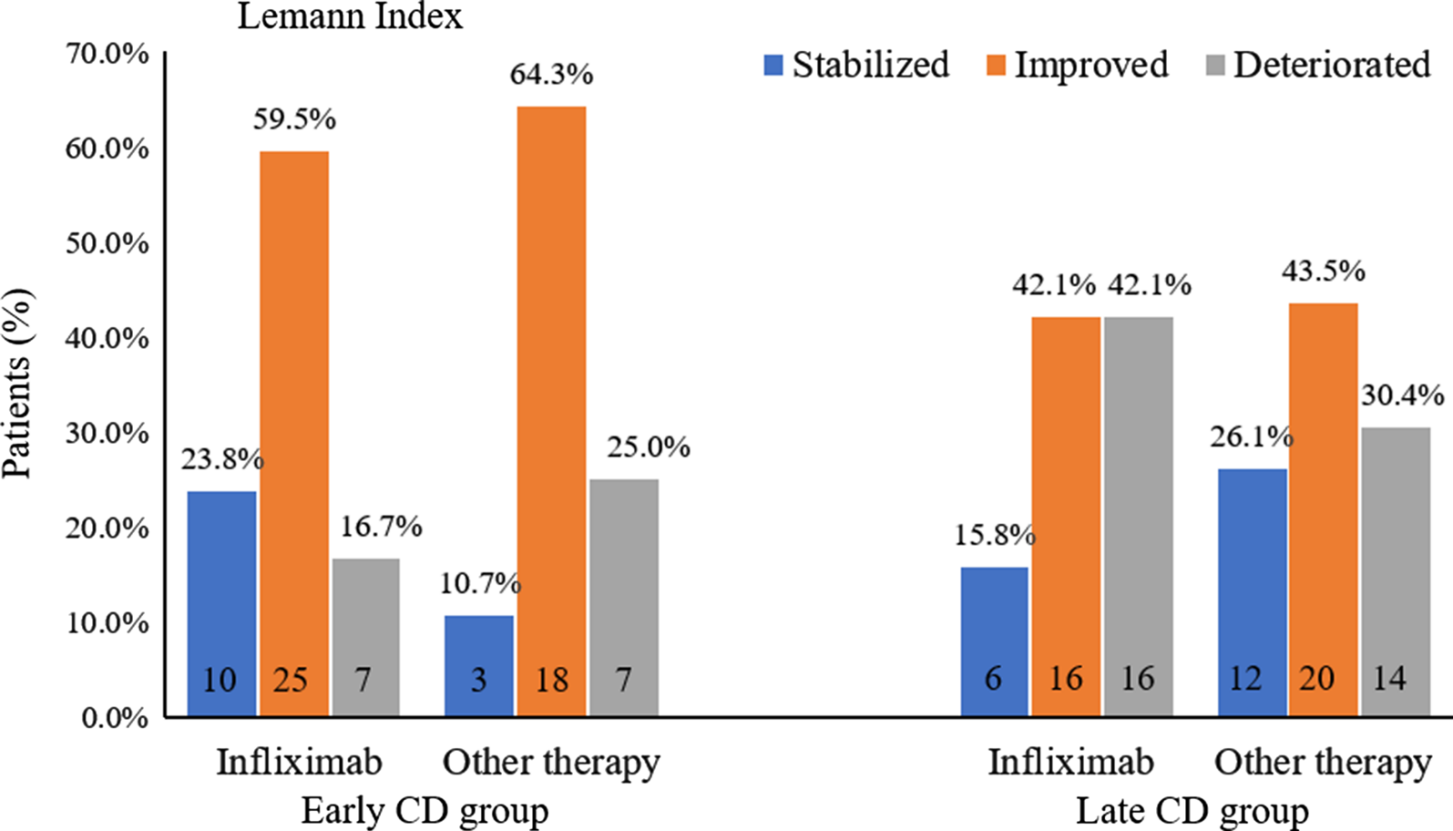
The effects of Infliximab and other therapy on Lémann Index (LI) of patients with different duration. Patients whose LI was unchanged were defined as “stabilized”, those with a decreased LI as “Improved”, and those with an increased LI as “Deteriorated”

Among the patients included in the early CD group, 42 patients received IFX therapy and 28 patients underwent other treatments, including azathioprine combined with steroids (n = 11), other immunomodulators (n = 9), enteral nutrition (n = 2), or mesalazine (n = 6). We found that IFX and other therapy could both reverse bowel damage in the early CD group (LI decreased from 5.5 [1.3–12.3] to 2.8 [0.3–7.4], *P* = 0.01 and from 6.9 [2.8–8.8] to 4.1 [1.4–8.0], *P* = 0.02, respectively). Twenty-five patients (59.5%) who received IFX and 18 patients (64.3%) who received other therapies experienced a decrease in LI during follow up. (*P* = 0. 59, Fig. 2). However, IFX induced a lower decrease in the LI score than other therapies (16.7% vs. 25.0%, *P* = 0.03).

In the late CD group, 38 patients received IFX treatment and 46 patients were treated with other drugs, including AZA combined with steroid (n = 14), other immunomodulators (n = 22), enteral nutrition (n = 7), or mesalazine (n = 3). Compared with other treatments, IFX did not present a major effect of improving BD (42.1% vs. 43.5%, *P* = 0.91), and more patients on IFX therapy experienced an increase in LI score compared with patients receiving other therapies (42.1% vs. 30.4%, *P* = 0.06, Fig. 2). The median LI scores were relatively stable in patients treated with IFX and in those treated with other drugs (Table 2).

**Table 2:** Different therapy in patients with early CD and late CD

Medical treatments	N	Inclusion LI	Follow up LI	*P* value
*Early CD*				
Total	70	6.3 [1.7–10.5]	3.4 [0.6–7.4]	0.001
IFX	42	5.5 [1.3–12.3]	2.8 [0.3–7.4]	0.01
Other therapy	28	6.9 [2.8–8.8]	4.1 [1.4–8.0]	0.02
Steroids + AZA	11	8.1 [6.5–12.8]	4.4 [1.8–11.0]	0.02
Immunomodulator	9	4.3 [1.8–7.2]	3.0 [1.8–6.3]	0.61
Enteral nutrition	2	11.0 [6.8–11.0]	7.4 [1.3–7.4]	0.18
Mesalazine	6	4.9 [0.3–8.1]	3.6 [0.3–7.5]	0.50
*Late CD*				
Total	84	5.9 [3.1–11.0]	5.2 [2.0–10.5]	0.71
IFX	38	6.6 [2.7–13.6]	6.7 [2.0–12.5]	0.93
Other therapy	46	5.5 [3.2–10.3]	4.3 [2.0–9.4]	0.20
Steroids + AZA	14	5.0 [3.7–6.5]	3.3 [2.3–8.4]	0.39
Immunomodulator	22	6.3 [2.9–11.4]	4.9 [2.3–9.3]	0.09
Enteral nutrition	7	10.6 [2.0–18.3]	2.4 [1.3–11.9]	0.71
Mesalazine	3	3.2 [1.0–4.9]	9.0 [1.0–13.2]	0.18

Data are expressed as median [IQR]

### Predictors of short-term intestinal resection in patients with CD

During the follow-up period, 22 patients (14.3%) underwent intestinal surgery, including 5 patients (7.1%) in the early CD group and 17 patients (20.2%) in the late CD group, respectively (*P* = 0.001). For patients receiving IFX therapy and other treatment, intestinal resection rates were not significantly different the early CD group (7.1% vs. 7.1%) and late CD group (21.1% vs. 19.6%).

**Fig. 3:**
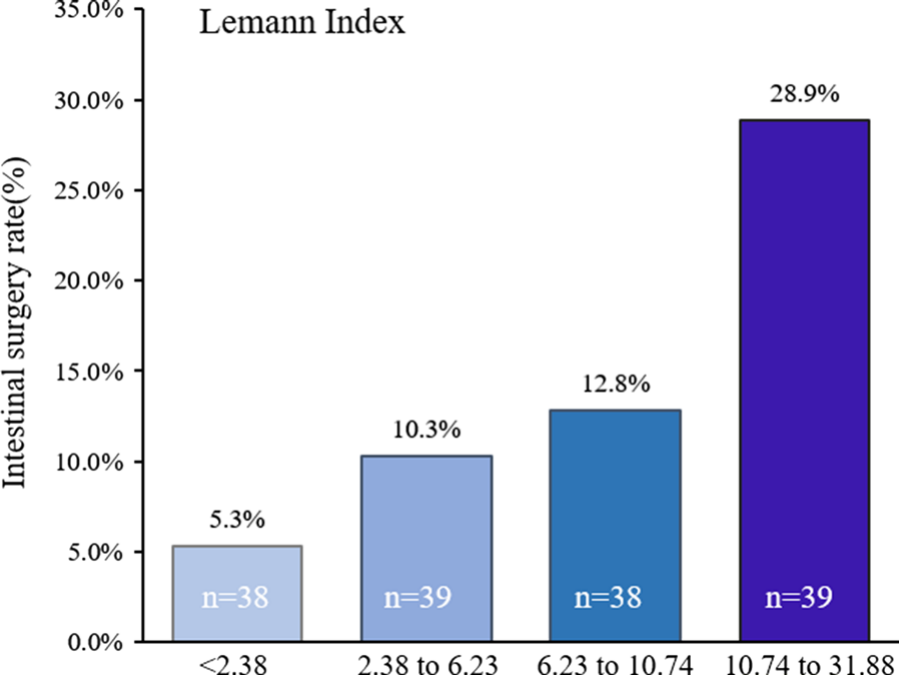
Quartile analysis of the Lémann index level for the rates of short-term intestinal resection in patients with Crohn’s disease

**Fig. 4:**
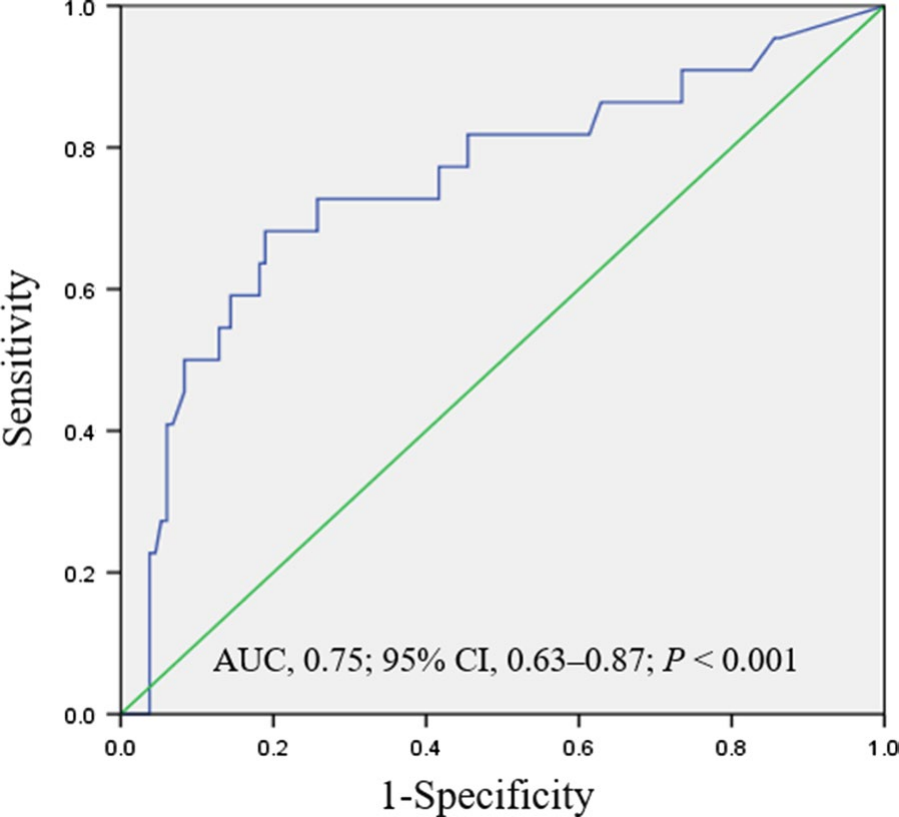
The receiver operating characteristic (ROC) analysis of the Lémann Index level to predict the risk of bowel resection in patients with Crohn’s disease. AUC: Area under the curve, 95% CI: 95% confidence interval

The relationship between the LI score and short-term intestinal resection rates was assessed by dividing LI into quartiles, and a significant LI level-dependent effect was observed on intestinal resection rates (5.3%, 10.3%, 12.8%, and 28.9%; *P* < 0.001) (Fig. 3). The cut-off value of the LI score to predict early bowel resection was determined using ROC analysis, which showed that the optimal cut-off threshold for the LI score was 8.99 (AUC, 0. 75, 95% CI, 0.63–0.87; *P* < 0.001) to discriminate the presence and absence of bowel resection, with a sensitivity of 68.2% and a specificity of 81.1% (Fig. 4).

The relationship between baseline LI levels and short-term bowel resection was further analyzed by dividing the LI scores into two groups (LI ≥ 8.99 and LI < 8.99). Univariate logistic regression analysis showed that, age at CD diagnosis (*P* = 0.09), disease duration (*P* = 0.03), early CD duration (*P* = 0.04), disease behavior B1 (*P* = 0.01), baseline LI > 8.99 (P = 0.008), and history of intestinal surgery (*P* = 0.001) were associated significantly with the early bowel resection in patients with CD. However, upon multivariate logistic regression analysis, only baseline LI > 8.99 (OR, 2.86; 95% CI: 1.05–7.83; *P* = 0.03), disease behavior B1 (OR, 0.20; 95% CI: 0.07–0.90; *P* = 0.04), and history of intestinal surgery (OR, 4.09; 95% CI: 1.43–11.74; *P* = 0.01), remained as significant independent risk factors for short-term bowel resection (Table 3).

**Table 3:** Predictors of the early bowel resection in patients with CD

Factors	Univariate analysis		Multivariable analysis	
	OR (95% CI)	*P* value	OR (95% CI)	*P* value
Age at CD diagnosis	1.03 (0.99–1.06)	0.09	1.01(0.96–1.06)	0.72
Disease duration, year	1.12 (1.01–1.24)	0.03	0.87 (0.45–1.68)	0.68
Early CD duration	0.40 (0.15–0.88)	0.04	0.67 (0.17–2.90)	0.39
Disease behavior (B1)	0.22 (0.07 -0.72)	0.01	0.20 (0.07–0.90)	0.04
Baseline LI > 7.99	3.55 (1.40–9.02)	0.008	2.86 (1.05–7.83)	0.03
History of intestinal surgery	5.64 (2.11–15.04)	0.001	4.09 (1.43–11.74)	0.01

## Discussion

In recent years, the Lémann index has been used frequently as a valid tool to monitor BD progression [11–13, 16]. The present study is the first to investigate the influence of different medical therapies on the LI score in consecutive patients with early CD, and identified predictors for short-term bowel resection. We included patients with data for at least two serial MREs and subdivided these patients into early and late CD groups according to the Paris classification. At present, the Paris classification is the most accurate method to define patients with early CD. We found no statistically significant difference in baseline LI scores between the two groups. After a median follow up of 17.0 months, in the early CD group, more patients experienced a decrease in the LI score (61.4% vs. 42.9%), whereas fewer patients showed an increase in the LI score (20% vs. 35.7%) compared with those in the late CD group. These findings indicated that patients with a shorter disease duration were less likely to have undergone BD progression compared patients with longer disease duration after medical treatment.

Recently, several cohort studies have been published that investigated the impact of early therapy on BD in patients with CD [17–20]. One study from Switzerland showed that early use of anti-TNF and/or immunomodulators in patients with CD within 24 months after diagnosis was related to a reduced risk of intestinal surgery and bowel strictures [17]. Another cohort study found that within 16 months after diagnosis, patients using anti-TNF and conventional therapy had similar levels of IBD-related complications [18]. However, these studies did not use the LI to evaluate the BD. The present study evaluated the effect of IFX and conventional therapy on BD using the LI score in patients with CD with different disease durations. We observed that IFX and other therapies both reversed BD in early CD patients (disease duration < 18 months). However, IFX was not more effective at improving BD for patients with longer disease duration. This result demonstrated the presence of a therapeutic window of opportunity to avoid irreversible BD, even though increasing evidence indicates that anti-TNF therapy could achieve higher mucosal healing and deep remission rates [21–23].

In this study, previous different previous intestinal surgery rates did not seem to affect baseline LI, whereas intestinal reception was one of the most decisive and scoring items in LI. During the follow-up period, the CD-related bowel resection rate was 14.3%, which was similar to certain previous studies, [24, 25] but lower than that in other studies [26, 27]. Patients with longer disease duration tended to have a higher intestinal surgery rate than patients with early CD (20.2% vs. 7.1%, *P* = 0.001). More importantly, patients receiving IFX did not suffer fewer CD-related intestinal resections compared with patients treated with other therapies. Then ROC analysis identified that an LI value of 8.99 was the optimal threshold to predict the risk of early intestinal resection. Other predictive factors for short-term intestinal resection were also assessed. We found that a higher risk of bowel surgery for CD was associated with a history of intestinal surgery, whereas disease behavior B1 could negatively predict the possibility of early bowel resection.

Several limitations of the present study should be acknowledged. First, its retrospective design might have the potential for selection bias and some possible confounders. Second, the choice of therapeutic strategies was affected partly by the patient's ability to pay. IFX, as the only biologic is approved in China, was not covered by the basic health insurance before January 1st 2020, which has led to a lower rate of IFX use [28]. Third, in our clinical practice, Chinese patients with CD have better responses to biological agents and are more likely to suffer myelosuppresion as the most common adverse effect when receiving concomitant immunomodulation; therefore, monotherapy with IFX alone is preferable and we didn’t include the few patients who received IFX combined with AZA. Finally, the duration of follow-up varied in this study and the median follow-up was only 17.0 months, which might have been insufficient for some subjects to develop to BD complications [29, 30]. We recommend that long-term outcomes be measured using the LI in further prospective study, even though several studies showed that BD may occur quite early [6, 31].

In conclusion, early intervention in CD could reverse BD, decrease the need for short-term intestinal resection, and change the natural history of the disease. In patients with CD, the LI is an effective and ideal measure to assess BD outcome. Moreover, patients with CD with a history of intestinal surgery at diagnosis, and/or a baseline LI > 8.99 should be treated aggressively and monitored carefully to prevent or block BD progression.

## Data Availability

The datasets used and analyzed during the current study available from the corresponding author on reasonable request.

## Abbreviations

CD: Crohn’s disease
BD: Bowel damage
LI: Lémann index
TNF: Anti-tumor necrosis factor
IFX: Infliximab
MRE: Magnetic resonance enterography
CDAI: Clinical disease activity scores
IQR: The interquartile range
ROC: Receiver operating characteristic
AUC: Area under the curve
CI: Confidence interval
